# Predictors of stigma in a sample of mental health professionals: Network and moderator analysis on gender, years of experience, personality traits, and levels of burnout

**DOI:** 10.1192/j.eurpsy.2019.14

**Published:** 2020-01-31

**Authors:** Marco Solmi, Umberto Granziol, Andrea Danieli, Alberto Frasson, Leonardo Meneghetti, Roberta Ferranti, Maria Zordan, Beatrice Salvetti, Andreas Conca, Silvia Salcuni, Leonardo Zaninotto

**Affiliations:** 1 Department of Neuroscience, University of Padova, 35128 Padova, Italy; 2 Neuroscience Center, University of Padova, 35128 Padova, Italy; 3 Department of General Psychology, University of Padova, 35131 Padova, Italy; 4 Department of Mental Health, AULSS 8 “Berica”, 36100 Vicenza, Italy; 5 Department of Mental Health, AULSS 6 “Euganea”, 35143 Padova, Italy; 6 Department of Mental Health, AULSS 2 “Marca Trevigiana”, 31100 Treviso, Italy; 7 Department of Mental Health, AULSS 7 “Pedemontana”, 36061 Vicenza, Italy; 8 Department of Mental Health, AULSS of Südtirol, Bolzano, Italy; 9 Department of Developmental Psychology and Socialisation, University of Padova, 35131 Padova, Italy

**Keywords:** Education in psychiatry, ethics and human rights, psychiatry in Europe, quality of care

## Abstract

**Background.:**

Stigma is one of the most important barriers to help-seeking and to personal recovery for people suffering from mental disorders. Stigmatizing attitudes are present among mental health professionals with negative effects on the quality of health care.

**Methods.:**

Network and moderator analysis were used to identify what path determines stigma, considering demographic and professional variables, personality traits, and burnout dimensions in a sample of mental health professionals (*n =* 318) from six Community Mental Health Services. The survey included the Attribution Questionnaire-9, the Maslach Burnout Inventory, and the Ten-Item Personality Inventory.

**Results.:**

The personality trait of openness to new experiences resulted to determine lower levels of stigma. Burnout (personal accomplishment) interacted with emotional stability in predicting stigma, and specifically, for subjects with lower emotional stability lower levels of personal accomplishment were associated with higher levels of stigma.

**Conclusions.:**

Some personality traits may be accompanied by better empathic and communication skills, and may have a protective role against stigma. Moreover, burnout can increase stigma, in particular in subjects with specific personality traits. Assessing personality and burnout levels could help in identifying mental health professionals at higher risk of developing stigma. Future studies should determine whether targeted interventions in mental health professionals at risk of developing stigma may be effective in stigma prevention.

## Introduction

According to social cognition theory, stigma is a multidimensional construct encompassing cognitive, emotional, and behavioral elements [1], and including the two dimensions of public stigma and self-stigma [2]. Public stigma reflects the attitudes and beliefs held by the general population and it could affect the daily interactions between the public and the individual suffering from mental disorders [3]. Self-stigma refers to the negative attitudes which those subjects turn against themselves, and it may have an effect on their personal experience with others and on their willingness to seek help [3–6].

Stigma is probably one of the most important barriers to help-seeking and to personal recovery [7,8]. Conversely, an inverse relationship has been found between stigmatizing attitudes and recovery orientation, in the sense that recovery oriented individuals may have less negative attitudes about people suffering from mental disorders [9]. Stigmatizing attitudes are widespread not only within health services in general but also in mental health facilities [10–14], with detrimental effects on the quality of health care received by the clients [8]. People suffering from mental disorders and/or substance use disorders have to face either an avoidant attitude by healthcare professionals [15] or prejudices about their adherence to medications [16], and about the “psychological” nature of their physical symptoms [17]. Some studies have shown that mental health professionals may have more negative views than the general public on stereotypes, restriction of the individual’s rights, and social distance [18,19]. The frequencies of discrimination reported by respondents to surveys about stigma range from 17% [20] to 31% [14] in a physical health-care setting and from 16% [13] to 44% [14] in a mental health-care setting. Negative staff attitudes have been linked with reluctance to use mental health facilities [4–6], poorer outcomes [21], and poorer customer’s satisfaction [22].

Professional burnout is considered as one of the most important factors explaining discrimination in mental health care [23,24], being common among mental health service providers and administrators [25–28]. Maslach et al. [29,30] described burnout as a construct including three dimensions: emotional exhaustion (EE), depersonalization (DP), and personal accomplishment (PA). The first refers to feelings of being depleted and fatigued, while DP refers to negative and cynical attitudes toward one’s work, and a reduced sense of PA (or efficacy) involves negative self-evaluation of overall job effectiveness [31].

Personality may also have a direct and moderating effect on generalized prejudice [32,33], since a significant negative relationships between some personality traits (i.e., openness and agreeableness) and prejudice has been found [34]. However, although the two concepts of prejudice and stigma may consistently overlap in their causes and consequences [35], a limited number of studies have explored a possible connection between personality and stigma in the proper sense [36–39].

The Five Factor model of personality [40] argues that each individual has five basic personality traits, namely, openness, conscientiousness, extraversion, agreeableness, and emotional stability. Higher levels of openness are typical of individuals who are considered highly sensitive, imaginative, curious, and open-minded. Conscientiousness refers to subjects who are supposed to be careful, efficient, and self-disciplined, while extraverted individuals are characterized by gregariousness, assertiveness, and dispositions toward positive emotions. Agreeableness is usually associated to people who are perceived as kind, sympathetic, cooperative, and tactful, while emotional stability refers to the individual’s ability to remain emotionally stable and balanced in front of a potentially difficult or harmful situation [41].

The current study aims to identify what path leads to stigma in a sample of mental health professionals, considering demographic and professional features, personality traits, and levels of burnout, by merging data-driven approach of network analysis [42–44] and moderator analysis.

## Methods

### Study design, participants, measures, primary, and secondary outcomes

The present study was conducted between July 2015 and December 2017 within six Community Mental Health Services (CMHS) operating in North-East Italy. The sample explored in a previous study was further extended [39]. The study protocol was first approved by the Research Ethics Committee of the Local Health Unit n. 8 (approval nr. 24091/8.2, 2015) and then extended to the other participating centers.

All the community mental health staff were invited to take part in the survey. Written informed consent was obtained from all participants. After completion of the questionnaire, anonymity was guaranteed by removing the face sheet including signed informed consent and personal identifiers (e.g., name and date of birth). Those who refused to join the study or did not sign informed consent were excluded. No personal or work experience information could be gathered about excluded subjects. Procedural details have been described and are available elsewhere [39]. The current sample was made of 318 mental health professionals including psychiatrists, psychologists, nurses, occupational therapists, and medical assistants (Table 1).

**Table 1.** Description of the sample including demographic features, personality traits, stigma, and burnout measures.

*n* = 318		Mean	%SD
Gender	Females	//	58.87
Age		46.37	8.23
Marital status	Single		18.48%
	Divorced		8.25%
	Married/cohabiting		71.28%
	Widowed		1.98%
Professional role	Nurse		64.82%
	Occupational therapist		1.95%
	Medical assistant		17.26%
	Psychiatrist		11.72%
	Psychologist		4.23%
Job setting	Inpatient		55.69%
	Outpatient		31.32%
	Rehabilitation		12.97%
Professional experience	Years	14.20	9.50
TIPI	Extraversion	3.93	1.53
	Agreeableness	5.64	0.93
	Conscientiousness	5.99	1.03
	Emotional stability	5.46	1.18
	Openness	4.89	1.25
AQ-9	Anger	1.23	1.24
	Avoidance	1.80	1.25
	Blame	1.41	0.95
	Coercion	6.55	2.39
	Dangerousness	3.57	1.86
	Fear	2.76	1.69
	Help	2.70	1.07
	Pity	4.84	2.17
	Segregation	1.42	1.14
	Total score	26.3	7.37
MBI	DP	2.87	3.50
	EE	15.48	8.28
	PA	32.18	9.70

Abbreviations: AQ-9, Attribution Questionnaire-9; DP, depersonalization; EE, emotional exhaustion; MBI, Maslach Burnout Inventory; PA, personal accomplishment; TIPI, Ten-Item Personality Inventory.

The primary objective of our study was to understand what may influence stigmatizing attitudes among mental health professionals, considering personality, professional experience, and levels of burnout, by means of a combination of network analysis and moderator analyses.

The following demographic and professional information were collected from participants: age, gender, years of education, marital status, professional role, years of work experience in the CMHS, and main place of employment within the CMHS (inpatient unit, outpatient unit, or rehabilitation unit). Then, to assess stigma, we used the Attribution Questionnaire-9 (AQ-9) [45], a brief version of the AQ-27 [46,47], a questionnaire developed by Corrigan on the basis on the Attribution Theory [48], which has been widely used in stigma research [49–52]. To assess personality traits, we used the Italian version of a brief instrument based on the Five Factor model of personality [40], the Ten-Item Personality Inventory (TIPI) [53,54]. The experience of burnout was measured using the Italian version of the Maslach Burnout Inventory (MBI) [55,56], which consists of 22 items measuring the three different components of burnout (EE, DP, and PA). Again, a detailed description of the instruments is available elsewhere [39].

### Network analysis

We examined a network composed of personality traits as measured by the TIPI, stigmatizing beliefs derived from the total score of the AQ-9, and burnout dimensions as measured by the MBI in a sample of mental health professionals. Network analysis has been performed with RStudio [57,58] using qgraph package as detailed elsewhere [43,58–63].

When using a network analysis approach to describe the complex interactions among a set of variables [58,64–66], these latter are represented as nodes, connected by edges, which are the visual representation of the correlation among nodes. In this perspective, nodes are reciprocally connected in a self-maintaining complex network of associations. However, network analysis does not allow any causal inference. More specifically, edges (correlations) of a network are undirected, and, unless longitudinal data are used, additional analyses must supplement network analysis to formulate any path or process hypothesis. With this network analysis, a graphical model of the network of included variables was built [59], as well as several properties of the estimated network were measured [67]. Since an excess of sparse correlations may constitute noise and would confuse rather than inform, we applied a penalty to correlations close to zero, in order to retain only meaningful associations. Such operation is also defined as a “least absolute shrinkage and selection operator” [68] regularization (a sort of shrinkage of small edges to zero), which was applied in order to only retain more solid *edges* (regularized partial correlations). Such regularization has been determined by setting Extended Bayesan Information Criterion [69], a parameter that sets the degree of regularization/penalty applied to sparse correlations to 0.5. We considered centrality indices of nodes that are relevant, since they describe how strongly the nodes are interconnected with several other nodes of the network. Centrality of nodes was estimated with node strength (i.e., the absolute sum of edge weights) [60]. As a proxy of reliability of a network’s estimates, we measured the stability of the network, by re-calculating centrality indices after dropping growing percentages of the included participants [58]. To quantify stability of the centrality indices, the correlation stability coefficient was calculated with a threshold of 0.25 indicating reliable stability. Finally, we measured edges’ accuracy by means of “nonparametric” bootstrapping (*n* boots = 1,000).

### Moderator analysis

Given that network analysis, together with the cross-sectional nature of the present study, does not allow any inference on causality or direction of the interconnections among nodes, we supplemented the network analysis with multiple regression and moderator analyses to identify stigma predictors among network’s nodes. We tested multiple regressions to analyze the influence of the level of burnout on stigma. After the visual inspection of the network, and in consideration of centrality indexes and of the correlation matrix, we identified three models (one for each dimension of burnout) to test what specific path could increase stigma in mental health professionals. We set the gender as a covariate. To obtain comparable coefficients, we mean-centered each predictor of the model; for gender, we used an orthogonal contrast coding that allows to compare, for each coefficient, the corresponding level of the factor to the average of the other levels. Whenever a significant interaction emerged, we computed a simple slope analysis: in particular, we refitted the original regression, centering the mediator to a standard deviation above and below its mean [70,71]. We fitted three multiple regression models, one per burnout dimension. Given the multiple testing, we decided to correct all the *p* values with the False Discovery Rate correction [72]. We computed all the statistical analysis by means of the R statistical software (http://www.r-project.org) version 2.10.0. Statistical analyses were run by U.G.

## Results

### Network analysis

Characteristics of included sample are reported in Table 1. Out of 318 questionnaires, we included 265 subjects without missing data in this network analysis.

**Figure 1.**
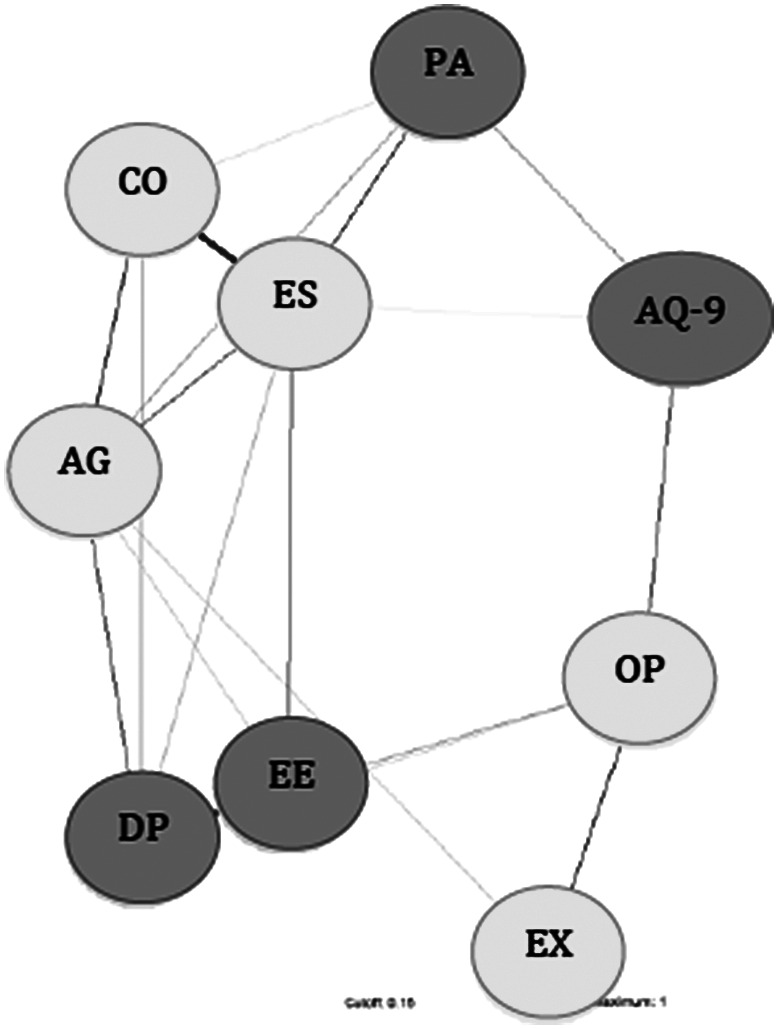
Network with personality traits, stigma, and burnout dimensions. Abbreviations: AG, agreeableness; AQ-9, Attribution Questionnaire-9; CO, conscientiousness; DP, depersonalization; EE, emotional exhaustion; ES, emotional stability; EX, extraversion; OP, openness; PA, personal accomplishment.

**Figure 2.**
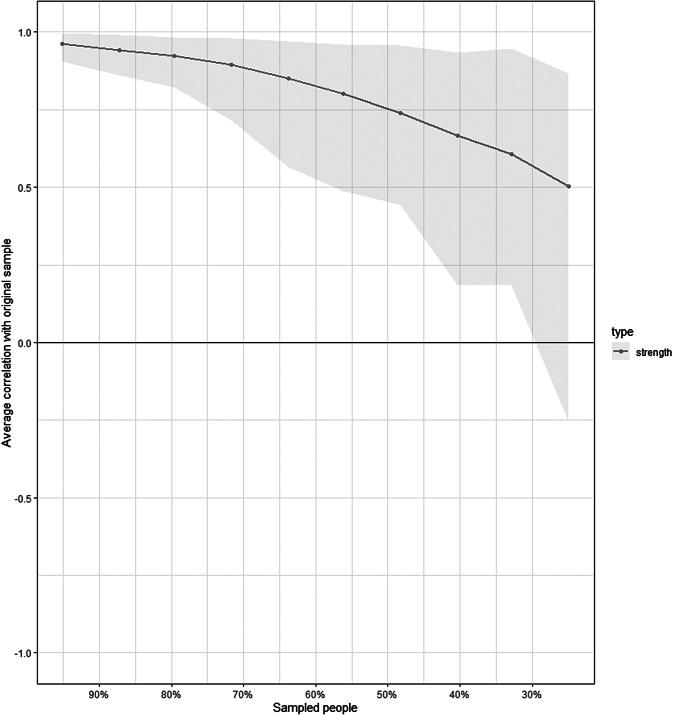
Stability of the network with progressively dropping proportions of the sample size.

The network is represented in Figure 1. Centrality estimates of the nodes of the network are reported in Table 2. Among personality nodes, emotional stability had the highest centrality. Given the small sample size, the network did not meet required stability indexes (stability coefficient = 0.24 vs. reliability thresholds = 0.25) as shown in Figure 2. At visual inspection of the network, openness was the only one directly connected with stigma. The burnout dimension of PA bridged emotional stability with stigma, while the other burnout dimensions were not directly connected with stigma. In Table 3, the correlation matrix of the network is also available, showing that the highest correlation values with stigma were for openness, emotional stability, and PA.

**Table 2.** Centrality indexes of the network

Node	Strength
AQ-9	
Total score	0.22
TIPI	
Extraversion	0.21
Agreeableness	0.58
Conscientiousness	0.55
Emotional stability	0.78
Openness	0.39
MBI	
EE	0.53
DP	0.56
PA	0.34

Abbreviations: AQ-9, Attribution Questionnaire-9; DP, depersonalization; EE, emotional exhaustion; MBI, Maslach Burnout Inventory; PA, personal accomplishment; TIPI, Ten-Item Personality Inventory.

**Table 3.** Network correlation matrix including personality, burnout, and stigma

	AQ-9	Extraversion	Agreeableness	Conscientiousness	Emotional stability	Openness	EE	DP	PA
AQ-9	1	−0.06	−0.05	−0.08	−0.13	−0.24	0.00	0.11	−0.19
Extraversion	−0.06	1	−0.14	−0.05	0.07	0.28	−0.06	−0.05	0.08
Agreeableness	−0.05	−0.14	1	0.38	0.34	0.06	−0.23	−0.30	0.22
Conscientiousness	−0.08	−0.05	0.38	1	0.46	0.01	−0.10	−0.23	0.21
Emotional stability	−0.13	0.09	0.34	0.46	1	0.06	−0.29	−0.25	0.33
Openness	−0.24	0.28	0.06	0.01	0.06	1	−0.18	−0.15	0.12
EE	0.001	−0.06	−0.23	−0.10	−0.29	−0.18	1	0.45	−0.04
DP	0.111	−0.05	−0.30	−0.23	−0.25	−0.15	0.45	1	−0.14
PA	−0.19	0.08	0.22	0.21	0.33	0.12	−0.04	−0.14	1

Abbreviations: AQ-9, Attribution Questionnaire-9; DP, depersonalization; EE, emotional exhaustion; PA, personal accomplishment.

### Multiple regression and moderator analysis

**Figure 3.**
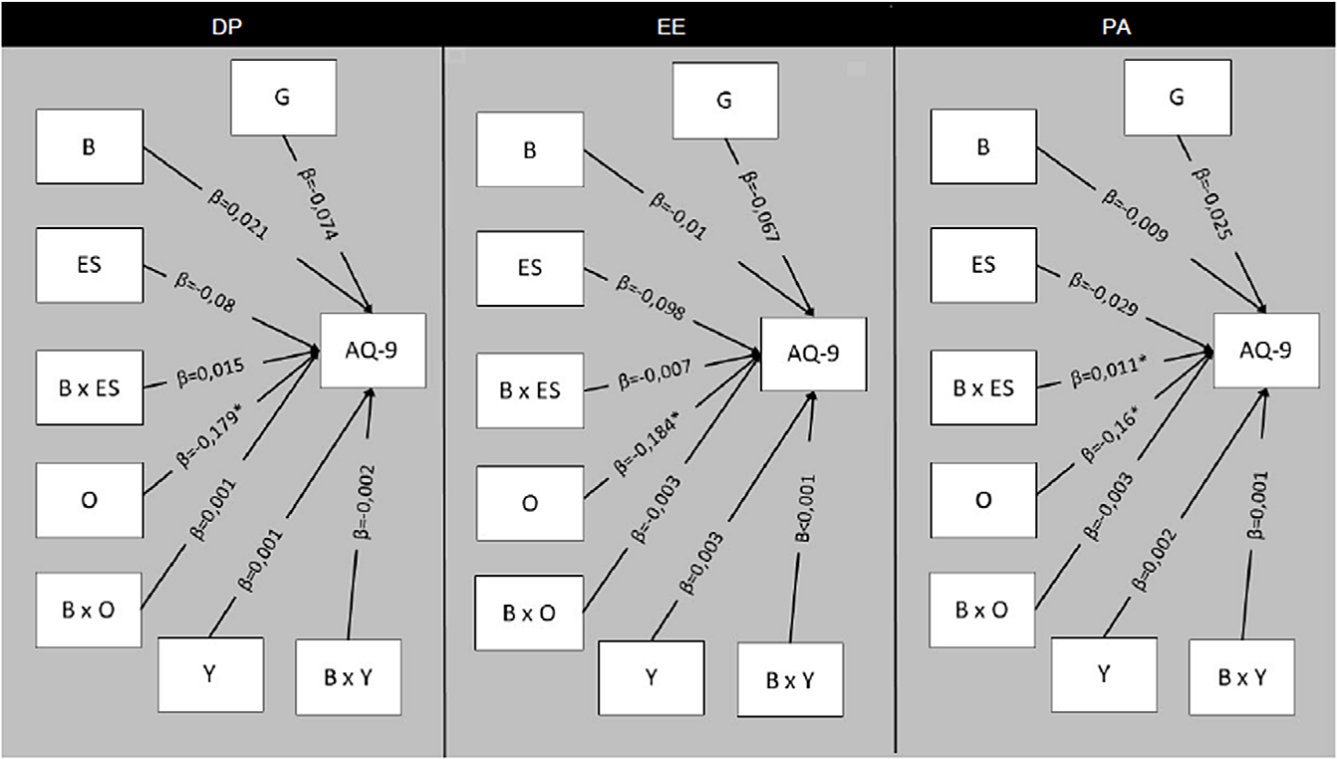
Design of the moderation models including predictors of stigma in a sample of mental health professionals. Abbreviations: *, significant predictors; AQ-9, Attribution Questionnaire-9; B, burnout as indicated by the header of each figure’s section (DP, depersonalization; EE, emotional exhaustion; PA, personal accomplishment); G, gender; ES, emotional stability; O, openness; X, indicates interaction; Y, years in psychiatry.

All the regressions’ result are displayed in Table 4 and represented in Figure 3.

**Table 4.** Results of the multiple regression model including predictors of stigma in mental health professionals

	DP					EE					PA				
	*β*	Std. error	*t*	*p*	*p* adj	*β*	Sth. error	*t*	*p*	*p* adj	*β*	Std. error	*t*	*p*	*p* adj
Gender (M)	−0.074	0.107	−0.689	0.491	0.737	−0.067	0.107	−0.624	0.533	0.749	−0.025	0.108	−0.234	0.815	0.880
Burnout	0.021	0.017	1.260	0.209	0.403	−0.010	0.007	−1.328	0.185	0.394	−0.009	0.006	−1.608	0.109	0.295
Emotional stability	−0.080	0.046	−1.731	0.085	0.254	−0.098	0.048	−2.046	0.042	0.142	−0.029	0.049	−0.592	0.555	0.749
Openness	−0.179	0.042	−4.209	<0.01	**<0.01**	−0.184	0.043	−4.266	<0.01	**<0.01**	−0.160	0.042	−3.819	<0.01	**0.001**
Years in mental health	0.001	0.006	0.186	0.853	0.885	0.003	0.006	0.491	0.624	0.802	0.002	0.005	0.403	0.687	0.807
Burnout × emotional stability	0.015	0.013	1.144	0.254	0.457	−0.007	0.005	−1.532	0.127	0.311	0.011	0.005	2.513	0.013	**0.049**
Burnout × openness	0.001	0.011	0.129	0.897	0.897	−0.003	0.004	−0.746	0.456	0.737	−0.003	0.004	−0.700	0.484	0.737
Burnout × years in mental health	−0.002	0.002	−1.315	0.190	0.394	0.000	0.001	0.415	0.678	0.807	0.001	0.001	−0.270	0.788	0.880

Bold *p*-values indicate significant predictors.

Abbreviations: DP, depersonalization; EE, emotional exhaustion; M, males; PA, personal accomplishment.

In the first model including DP, we did not find neither direct nor moderating effect of this dimension of burnout on stigma. However, we found that openness inversely predicted stigma (*β* = −0.179; *p* adjusted <0.001) without any moderating effect of other variables.

In the second model, we did not find neither direct nor moderating effect of EE on stigma, but openness was confirmed as an inverse predictor for stigma (*β* = −0.184; *p* adjusted <0.001), without any moderating effect of other variables.

Finally, considering PA as a predictor, we confirmed that higher openness scores directly led to a lower negative attitude (*β* = −0.16; *p* adjusted = 0.001). Moreover, we found a significant interaction between PA and emotional stability (*β* = 0.011; *p* adjusted = 0.049) in affecting stigmatizing attitudes. In particular, we found that the less the worker felt a sense of efficacy (low PA), the more negative was his/her attitude toward patients, an effect which became significant only when the individual reported lower scores on emotional stability (*β* = −0.023; *p* adjusted = 0.002).

In all three models, neither gender nor years of experience predicted anyhow (directly or via moderators) stigma.

## Discussion

The present study confirmed previous findings by our group [39] on an association between personality and stigma. In our sample, the personality trait of openness resulted to have a relevant effect on the development of stigmatizing attitudes among mental health professionals. Moreover, higher levels of burnout (low PA) were associated to more negative views about clients, in particular in those subjects showing a lower emotional stability.

These findings can have simple but relevant implications for the organization of mental health facilities. First, by pointing at the importance of individual differences on the development of negative attitudes toward patients, they suggest that it may be necessary to consider these differences when addressing the problem of stigma among mental health professionals, especially in the earlier stages of education. Our results are in line with previous studies exploring samples of college students [36] and healthcare students [37], showing a negative association between stigma and two dimensions of personality, namely agreeableness and openness. Those features may be accompanied by better empathic and communication skills [73], which in turn may affect the type of contact with the patient. In our sample, openness resulted to have a direct effect on stigma. Openness is characteristic of individuals who are more flexible, reflective, sensitive, and imaginative [41]. People scoring higher on openness may be more prone to develop positive contact experiences, having a better disposition toward understanding the feelings of individuals suffering from mental disorders. Moreover, individuals with higher levels of openness may be more prone to a positive and recovery-oriented attitude, which in turn has been associated to lower levels of stigma [74,75].

The second important result is that not all burnout dimensions, but only low PA in conjunction with low emotional stability may have a relevant effect on stigma. Stigma has been consistently associated with burnout among mental health professionals [23,24], to the point that some authors [76] have also argued that some negative attitudes toward patients may be one of the emotional aspects of burnout. Burnout in mental health professionals has been linked to workload, role conflict, lack of job control, and a reduced sense of autonomy at work [77]. In our previous study [39], higher levels of PA were associated to the presence of institutional responses to risk situations (namely, protocols for managing the aggressive or violent behaviors).

A high PA is usually regarded not only as the sense of efficacy and effectiveness of one’s personal resources, but also as the sense of involvement and commitment to one’s job [78], a characteristic which may in turn have an effect on the tendency to engage into positive contacts with patients. Conversely, a high emotional stability is connected to a strong sense of ability to control events, and may act as a protective factor against burnout [39,79], by facilitating the employment of better coping strategies such as problem-solving and cognitive restructuring [80]. Thus, a stronger sense of control, deriving from both personality traits and from workplace’s characteristics, may act as a protective factor against negative attitudes toward patients.

The present work has several limitations. First, the small sample size did not allow to run more fine-grained network analysis and resulted in an unstable network, whose results should be interpreted from a descriptive and qualitative perspective. Second, the present study is cross-sectional and based on self-reported and anonymous measures, which may be affected by some response bias toward social desirability. However, the use of two different statistical techniques may to a certain degree compensate the cross-sectional design of the study and may reveal a direction of effect from personality to burnout to stigma.

In conclusion, mental health professionals having low openness may be more prone to develop stigma, and burnout increases stigma in particular in subjects with lower emotional stability. Mental health service organizations should consider implementation of personality and burnout assessments to promptly intervene and minimize stigma among mental health professionals. Further, as suggested by Yuan et al. [37], since personality traits continue to develop, especially during young adulthood, future studies should address the role of personality when testing antistigma interventions, especially when they are directed to early stages of education of future healthcare professionals. Finally, given the importance of recovery orientation for stigma research [81–83], future studies should address the issue of the relationship among personality traits, stigmatizing beliefs, and recovery orientation among mental health professionals.

## Financial Support

This research did not receive any specific grant from funding agencies in the public, commercial, or not-for-profit sectors.

## Conflict of Interest

The authors declare no conflict of interest.
